# Biological Response of Planktic Foraminifera to Decline in Seawater pH

**DOI:** 10.3390/biology11010098

**Published:** 2022-01-09

**Authors:** Shuaishuai Dong, Yanli Lei, Hongsheng Bi, Kuidong Xu, Tiegang Li, Zhimin Jian

**Affiliations:** 1 Laboratory of Marine Organism Taxonomy and Phylogeny, Center for Ocean Mega-Science, Institute of Oceanology, Chinese Academy of Sciences, Qingdao 266071, China; dongshuaishuai@qdio.ac.cn (S.D.); kxu@qdio.ac.cn (K.X.); 2 Southern Marine Science and Engineering Guangdong Laboratory (Zhuhai), Zhuhai 519082, China; 3 University of Chinese Academy of Sciences, Beijing 100049, China; 4 Chesapeake Biological Laboratory, University of Maryland Center for Environmental Science, Solomon, MD 20688, USA; hbi@umces.edu; 5 Key Laboratory of Marine Sedimentology and Environmental Geology, First Institute of Oceanography, Ministry of Natural Resources, Qingdao 266061, China; 6 State Key Laboratory of Marine Geology, Tongji University, Shanghai 200092, China

**Keywords:** biological response, *Trilobatus sacculifer*, seawater pH, symbiont-bearing calcifiers, bleaching, on-board experiment

## Abstract

**Simple Summary:**

The ocean absorbs large amounts of CO_2_ emitted by human activities, which leads to a decrease in seawater pH, and has the potential to cause damage to calcareous marine organisms. Planktic foraminifera are some of the most important calcareous marine organisms in the ocean, although the biological response of planktic foraminifera to the decline in seawater pH is still unknown. In this study, the biological response of planktic foraminifera to declining seawater pH was studied through a series of on-board experiments. The experimental results showed that the decrease in seawater pH adversely affected the biological processes of planktic foraminifera, resulting in weaker predation, slower growth, lighter shells, and more deformities. In addition, for the first time, we report that microalgae that live with planktic foraminifera were also killed under low-pH conditions. Several indices were established to quantify the relationships between the biological parameters of planktic foraminifera and seawater pH, which could be used to reconstruct the paleoceanographic seawater pH. This study provides experimental data to quantify the biological response of calcareous plankton to a decline in seawater pH.

**Abstract:**

Understanding the way in which a decline in ocean pH can affect calcareous organisms could enhance our ability to predict the impacts of the potential decline in seawater pH on marine ecosystems, and could help to reconstruct the paleoceanographic events over a geological time scale. Planktic foraminifera are among the most important biological proxies for these studies; however, the existing research on planktic foraminifera is almost exclusively based on their geochemical indices, without the inclusion of information on their biological development. Through a series of on-board experiments in the western tropical Pacific (134°33′54″ E, 12°32′47″ N), the present study showed that the symbiont-bearing calcifier *Trilobatus sacculifer*—a planktic foraminifer—responded rapidly to a decline in seawater pH, including losing symbionts, bleaching, etc. Several indices were established to quantify the relationships between these biological parameters and seawater pH, which could be used to reconstruct the paleoceanographic seawater pH. We further postulated that the loss of symbionts in planktic foraminifera acts as an adaptive response to the stress of low pH. Our results indicate that an ongoing decline in seawater pH may hinder the growth and calcification of planktic foraminifera by altering their biological processes. A reduction in carbonate deposition and predation could have profound effects on the carbon cycle and energy flow in the marine food web.

## 1. Introduction

Symbiont-bearing calcifiers, including planktic foraminifera, are sensitive to a decline in seawater pH [1,2]. Planktic foraminifera are ideal model organisms for use in studies on the effect of pH [3,4,5], and existing studies show that they are adversely affected by a decline in pH, i.e., through a lowering of shell weight, a reduction in individual size, etc. [6,7,8]. Most of the previous studies focused on geochemical proxies, such as the elemental and isotopic composition of the foraminifer shells [9,10,11,12]. However, geochemical analysis often requires a large number of planktic foraminiferal samples [13], limiting its application in areas with low numbers of planktic foraminifera, such as oligotrophic regions [14,15]. There is a clear need to develop alternative indices to evaluate the impact of a declining pH on these organisms—especially in oligotrophic regions, which cover most of the earth.

The planktic foraminifer *Trilobatus sacculifer* is an extant species with a long evolutionary history of ~23 million years [16]; it hosts dinoflagellate symbionts (zooxanthellae) [17,18], and is widely distributed in tropical and subtropical surface waters—especially in oligotrophic environments [19]. However, the biological response of symbiont-bearing planktic foraminifera to a decline in seawater pH has rarely been studied, e.g., in terms of survival, growth, calcification, and symbiotic activity. Furthermore, the lack of information on the biological response of symbiotic plankton to a decline in seawater pH limits the application of biotic indices in reconstructing the pH of ancient oceans [10].

In the present study, we quantified the effects of a decline in pH on planktic foraminifera in an oligotrophic environment, using culture experiments. Newly collected live *T. sacculifer* from the western tropical Pacific (134°33′54″ E, 12°32′47″ N) were cultured on-board and under controlled pH conditions, in order to quantify their biological response. Based on the experiments, we examined the relationships between seawater pH and biological parameters (e.g., growth, length, weight)—which are useful tools in reconstructing the paleoceanographic pH—and verified the role of pH in the evolution of symbiont-bearing calcifiers.

## 2. Materials and Methods

### 2.1. Sample Collection

Live *T. sacculifer* were collected using a plankton net (76 μm mesh size and 0.1 m^2^ mouth area) that was towed vertically from a 200 m depth to the surface, and were then incubated on-board in the western tropical Pacific (134°33′54″ E, 12°32′47″ N, 3281 m) in December 2015 during a Yap Seamount survey cruise of the R/V KEXUE. The water column temperature and salinity in the upper 200 m were measured with a Seabird CTD probe, and the results are presented in Figure S1A,B and Table S1. The in situ light intensity (range: 2517.42–1.02 μmol photons m^−2^ s^−1^ from a 5.1 to 200 m depth) was measured using Hyper-Profiler II (Satlantic Inc., Halifax, NS, Canada), the results of which are presented in Figure S1C and Table S1. The surface seawater pH was measured using a pH meter (Mettler Toledo Delta 320, Mettler-Toledo Instruments (Shanghai) Co., Ltd., China, with a precision of 0.01 unit), and was ~8.10. Based on the temperature and salinity curve and the study by Palmer et al. [20], the ocean pH ranges from ~8.10 to ~7.50 at a depth between 0 and 200 m in the equatorial Pacific region. Therefore, we set the experimental seawater pH gradient as 7.20–8.65, which includes the seawater pH range within the depth of 0–200 m. This selected seawater pH range also reflects the predicted future pH decline in 2100 and 2300 (~7.70–7.40), and could be used to explore the effects of extreme high-(8.65) and low (7.20)-pH conditions.

### 2.2. Seawater Preparation

In situ seawater, filtered by 0.22 μm glass microfiber filters, was used for the culture experiment, and the pH of seawater was altered by adding 0.1 mol/L HCl or 0.1 mol/L NaOH (NaOH was freshly prepared prior to use) to ranges of 7.20–8.40 and 7.40–8.65, respectively. The pH measurements were conducted using a pH meter (Mettler Toledo Delta 320, with a precision of 0.01 unit). The pH sensor was three-point calibrated with standard buffer solutions of pH = 4.01, pH = 7.00, and pH = 10.01 at 25 °C before measurement.

### 2.3. Incubator Parameter Setting

During the cruise, we carried out two sets of culture experiments for planktic foraminifera, including an individual experiment for *T. sacculifer* individuals and a community culture experiment for the *T. sacculifer* population. All experiments were carried out in an incubator (SANYO MLR-351 H, SANYO Electric Co., Ltd., Osaka, Japan) equipped with R/V KEXUE. There were six levels of light intensity (LS0–LS5) for the incubator. The maximum level of light intensity available (LS5, ~170 μmol photons m^−2^ s^−1^/20,000 Lux) and a 12 h:12 h light: dark cycle at 27 °C (Figure S2) were used. The maximum daylight illumination measured on site was ~2500, 400, 50, 5, and 1 µmol·m^−2^·s^−1^ at depths of 5, 50, 100, 150, and 200 m, respectively. Juvenile foraminifera mostly reside at a depth of 50–100 m, and there are less symbionts in the juvenile stages than in adults. We selected the required light intensity for juvenile growth, given that we were interested in the juvenile stages of growth. Additionally, previous culture experiments showed no considerable differences in individual growth between high and low light intensity ([17] and references therein), which also confirms that the light intensity used in this experiment was reasonable.

### 2.4. Individual Experiment Setting

Living *T. sacculifer* individuals were identified and selected from the water sample on-board using a stereomicroscope immediately after collection. The selected individuals were incubated in a beaker with seawater at 27 °C. The adults were around 300–400 μm in length in this region, and juveniles were 200–300 μm. Given that the objective of the present study is to investigate the biological changes, juveniles of a relatively small size (~200 μm) were selected for the experiments.

The detailed description of the individual experiment is presented in Table S2. To avoid any sudden experimental disturbances resulting from the *T. sacculifer* being transferred directly to the intended pH, the pH was adjusted gradually. In this experiment, the pH was set at 5 different levels—7.20, 7.50, 7.80, 8.10, and 8.40—with 4–5 replicates at each level for a 10-day incubation period. To observe the daily growth of each individual and to avoid the entanglement of pseudopodia, individuals were placed separately in a 150 mL sealed borosilicate bottle filled with seawater (see Figure S2B, explaining that this can effectively reduce the exchange between the CO_2_ in the solution and atmosphere). Each individual was fed a living 1-day-old *Artemia nauplius* every day. *Artemia nauplius* is a brine shrimp widely used in culturing planktic foraminifera [11,17], and it was incubated on board during the experiment. Each *Artemia nauplius* was transferred using a pipette and released ~1 cm away from the *T. sacculifer* under a stereoscope. During incubation, the seawater in each bottle was replaced by half-freshly-filtered seawater, with the pH pre-adjusted to predetermined levels every day.

### 2.5. Community Experiment Setting

Water samples containing planktic foraminifera were randomly divided into 6 even groups, which were cultured in 2000 mL closed borosilicate jars filled with seawater of different pH levels (7.40, 7.65, 7.90, 8.15, 8.40, 8.65) for 12 days of incubation. To distinguish the growing individuals under controlled conditions, the fluorescent calcite marker calcein (5 mg/L) was added to the culture seawater for the whole experiment. We used the same procedure for pH treatments, feeding, and seawater replacement as described in a previous experiment.

### 2.6. Shell Analysis

In the individual experiment, the shell diameter (i.e., the length of the shell, from top to bottom) of each living *T. sacculifer* was measured daily. Meanwhile, individuals that sank to the bottom with no sign of pseudopodia movement under a stereoscope were considered “dead”, and were removed from the experiment. The growth rate (μm/day) of each individual was calculated by dividing the length of growth by the number of days survived.

In the community experiment, all 6 groups were sieved over a 63 µm sieve to remove excess seawater at the end of the 12-day incubation. The deposition retained on the 63 µm sieve was then fixed using 95% alcohol. All specimens were then placed onto a gridded slide and examined under a fluorescence microscope (Olympus BX53, Olympus Corporation, Tokyo, Japan, blue excitation, 470–495 nm). Only the fluorescent *T. sacculifer* specimens were counted and measured, and a total of 39 specimens were obtained (see Figure S3). The size of each *T. sacculifer* was measured under Olympus SZX16 stereomicroscope using cellSens Standard software (Olympus Corporation, Tokyo, Japan).

Specimens of *T. sacculifer* were placed in a sodium hypochlorite bath to remove the remaining organics. The specimens were rinsed 4 times with deionized water and then oven-dried at 50 °C. Finally, each specimen of *T. sacculifer* was weighed using an ultra-microbalance (Sartorius CP2P, precision = 1 μg), and the average shell weight for the group was calculated by dividing the sum of the measured weights by the total number of whole foraminiferal shells. Additionally, a new biotic index was calculated to evaluate the normalized shell weight using the ratio between the weight and length of individual foraminifera (W/L = weight/length). The W/L was similar to the SNW (size-normalized weight), but was more straightforward than the latter [21]. To calculate the SNW, the test silhouette area must be measured and the test diameter calculated. As to the W/L, the length could be measured directly. Thus, the W/L is more convenient and practical than SNW.

### 2.7. Statistical Analyses

Mean survival numbers and estimated life expectancy were estimated using life table methods modified from the work of Gerasimova et al. [22], and were calculated using all of the data from the individual experiments. To test the effects of pH on individual survival time, growth rate, shell length, and weight, a one-way ANOVA was performed using the Statistical Analysis System (SAS, SAS Institute Inc., Cary, NC, USA). All of the above data were log(x + 1) transformed to meet the assumptions of normality. The homogeneity of variances was tested with Levene’s test (significant effects of pH). One exception was that, due to the unsatisfactory normality and/or homogeneity of variances, differences in the survival time and growth rate of *T. sacculifer* were tested using a nonparametric test (Kruskal–Wallis test in SAS). Duncan’s multiple range test was used to check for differences between groups (α = 0.05), and differences were considered significant at *p* < 0.05. To establish the potential correlations between pH and biological parameters (i.e., individual growth rate, shell length, weight, and W/L), a correlation and regression were conducted using the OriginPro 9.0 software packages (OriginLab Corporation, Northampton, MA, USA). The raw data were used in the above analysis.

## 3. Results

### 3.1. Biological Parameters from Individual Experiments

The results of the individual experiments showed that pH had a significant effect on the survival time of *T. sacculifer* (*df* = 4, *F* = 9.76, *p* < 0.0001). The estimated survival time was longer for pH = 8.40 and pH = 8.10 than for pH = 7.80, pH = 7.50 and pH = 7.20 (Figure S4A). The survival time was higher at pH = 7.80 than at pH = 7.20. The estimated mortality was high and life expectancy was short, i.e., a low survival rate (inverse of life expectancy) was observed in the low-pH treatments (Table S3, Figure S5).

**Figure 1:**
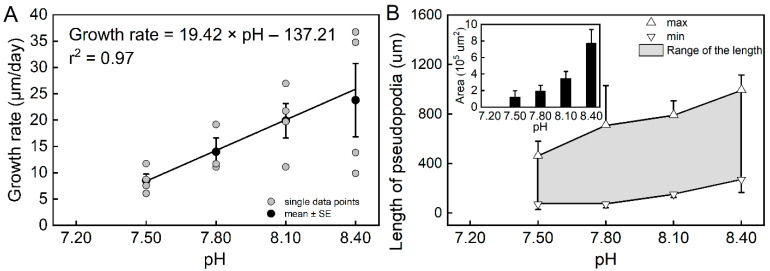
The relationship between the growth rate of *Trilobatus sacculifer* and seawater pH (**A**), and the variations in pseudopodia of *T. sacculifer* under different pH treatments (**B**). Note: All *T. sacculifer* individuals cultured at pH = 7.20 died within the first day, and the growth rate could not be calculated or analyzed for regression. Error bar = SE. The relatively high variability in the treatments of pH = 8.10 and pH = 8.40 could be a result of the relatively small sample sizes.

The results of the individual experiments showed that pH had a significant effect on the growth rate of *T. sacculifer* (*df* = 3, *F* = 4.39, *p* = 0.0196). The estimated growth rates were higher at pH = 8.40 and pH = 8.10 than at pH = 7.50 (Figure S4B). The estimated growth increased sharply from pH = 7.50 to pH = 8.10, but was found to be rather slow from pH = 8.10 to pH = 8.40, suggesting that the low pH caused a sharp decrease in *T. sacculifer* growth. All *T. sacculifer* individuals that were cultured at pH = 7.20 died within the first day, which also suggests a negative impact of low pH. A correlation analysis suggested that the estimated growth rates were highly correlated with pH (r^2^ = 0.97, *n* = 4, *p* = 0.01468, Figure 1A).

During the individual experiments, we observed the movement and feeding of individual planktic foraminifera. Compared with the reference pH (pH 8.10) and high pH (8.40), planktic foraminifera had reduced locomotion and feeding capacity in the low-pH treatments. The length and the cross-sectional area covered by pseudopodia were used to quantitatively evaluate the movement ability. Both the length and area of pseudopodia decreased as the pH declined (Figure 1B). Additionally, the range in length reduced under the low-pH treatments, indicating less flexibility. Furthermore, a similar result was observed for feeding behavior. The *T. sacculifer* with long and luxuriant pseudopodia were more capable of catching and ingesting the *Artemia nauplius*. In contrast, individuals under the low-pH treatments had a reduced ability, or lost the ability, to catch the *Artemia nauplius*.

**Figure 2:**
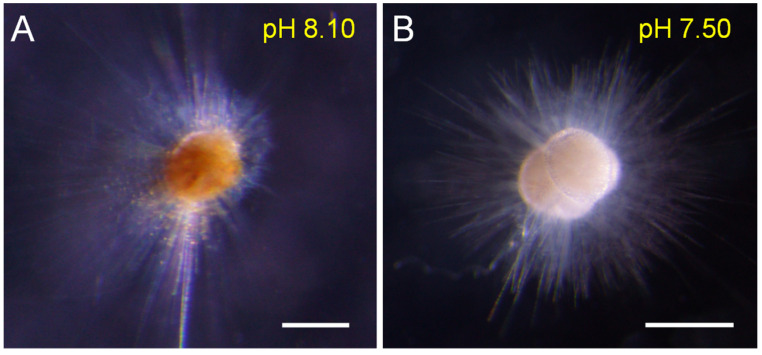
The color comparison between different individuals under different pH conditions shows the bleaching phenomenon under low-pH conditions (**A**): the normal individual; (**B**): the bleached individual). Scale bar = 200 μm.

**Figure 3:**
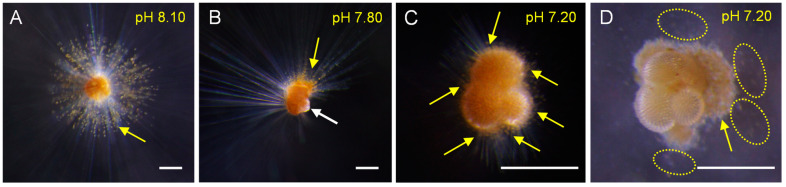
Variations of symbionts in living *T. sacculifer* under different pH conditions. (**A**) Many symbiotic dinoflagellates were dispersed among their long pseudopodia under the pH 8.10 conditions (the brown particles identified by the yellow arrows are symbiotic dinoflagellates, i.e., zooxanthellae). (**B**) Only a small number of symbiotic dinoflagellates gathered at the roots of the pseudopodia under the pH 7.80 conditions. The yellow arrow shows the gathered symbionts, while the white arrow shows the loss of pseudopodia and the partly empty chamber. (**C**,**D**) Loss of symbiotic dinoflagellates in a living *T. sacculifer* from the same specimen under the pH 7.20 conditions. At first, the symbiotic dinoflagellates were distributed around the shell, as indicated in panel **C**, with yellow arrows indicating the symbiotic dinoflagellates. Then, the pseudopodia and symbiotic dinoflagellates gradually disappeared, and some of the remaining symbionts gathered together, as indicated in panel **D**, with the yellow arrow showing the gathered symbiont, while the yellow circles show the symbionts that separated from the host. Scale bar = 200 μm.

The cytoplasmic color could reflect individual vitality. Our results showed that the cytoplasmic color became lighter and whiter under low-pH conditions (<7.80), and was darker and brighter under the reference and high-pH treatments (Figure 2). Symbionts also gradually disappeared in low-pH (<7.80) seawater (Figure 3). Under normal conditions (e.g., pH = 8.10), many symbiotic dinoflagellates were dispersed among their long pseudopodia. When the pH decreased (e.g., pH = 7.80), only a small number of symbiotic dinoflagellates gathered at the roots of the pseudopodia. Under more acidic conditions (e.g., pH = 7.20), all of the pseudopodia and symbiotic dinoflagellates disappeared.

### 3.2. Biological Parameters from the Community Experiment

**Figure 4:**
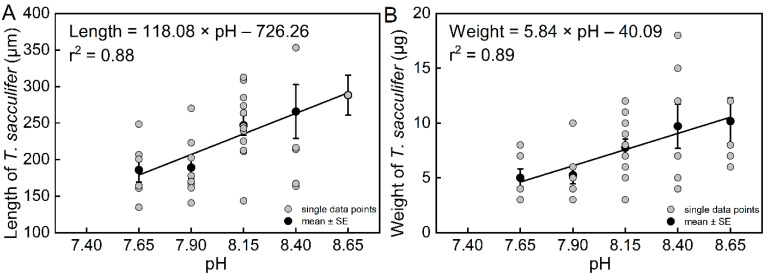
The relationships between length (**A**) and weight (**B**) of *T. sacculifer* versus seawater pH. Note: There were no living *T. sacculifer* under the pH 7.40 treatment, and data from this treatment were not analyzed for regression. Error bar = SE. Similar to the growth rate in the previous section, there was high variability due to the relatively small sample sizes.

The results of the community culture experiment show that pH had a significant effect on the length of *T. sacculifer* (*df* = 4, *F* = 4.15, *p* = 0.0077). The final lengths of *T. sacculifer* at pH = 8.65, 8.40, and 8.15 were higher than their lengths at pH = 7.90 and 7.65 (Figure S6A). The length of *T. sacculifer* was highly correlated with pH (Figure 4a, r^2^ = 0.88, *n* = 4, *p* = 0.0181). The final lengths of *T. sacculifer* cultured for 12 days at all levels of pH are provided in Table S4.

**Figure 5:**
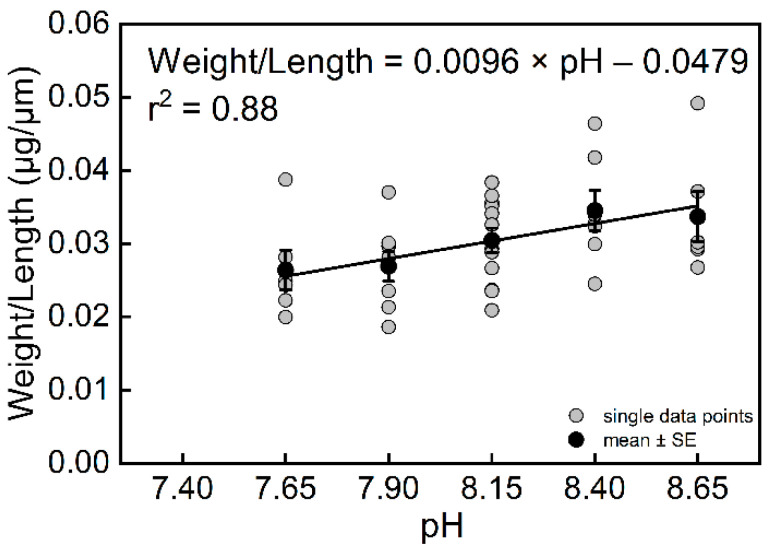
The relationship between weight/length of *T. sacculifer* and seawater pH. Note: There were no living *T. sacculifer* under the pH 7.40 treatment, and data from this treatment were not analyzed for regression. Error bar = SE.

The results also showed that pH had a significant effect on the weight of *T. sacculifer* (*df* = 4, *F* = 3.43, *p* = 0.0184). The final weights of *T. sacculifer* at pH = 8.65 and 8.40 were higher than at pH = 7.90 and 7.65 (Figure S6B). The weight of *T. sacculifer* was highly correlated with pH (Figure 4B, r^2^ = 0.89, *n* = 4, *p* = 0.0159). The final weights of *T. sacculifer* after being cultured for 12 days at all levels of pH are provided in Table S5. In the present study, we calculated the ratio between weight and length as a new biotic index, which represented the normalized shell weight, and was also found to be highly correlated with pH (Figure 5, r^2^ = 0.88, *n* = 4, *p* = 0.0186).

## 4. Discussion

In this study, we carried out qualitative observations and quantitative measurements to explore the relationships between planktic foraminiferal biological parameters and seawater pH. Compared with previous studies, this study provides a comprehensive evaluation of the biological response of living planktic foraminifera to changes in seawater pH. The biological response of planktic foraminifera to changes in pH included growth, feeding, vital rates, and calcification.

### 4.1. Biological Response of T. sacculifer to pH Decline

To date, there have only been a few studies focused on the response of planktic foraminifera to temperature, salinity, and nutritional conditions [23,24], without information on seawater pH. Our study provides the first results documented on the responses of planktic foraminiferal biological processes to seawater pH. We identified the biotic indices (i.e., growth, length, weight, and W/L) that are clearly affected by seawater pH based on the culture experiments (Table 1). The results offer alternative and sensitive indices different from the geochemical approach, and which require fewer samples.

The size of planktic foraminifera is one of the biggest concerns for scientists, especially in studies using sediment cores [25,26]. Change in size may act as an indicator of seawater conditions. In the present study, the growth rate declined as the pH declined, and the final length of *T. sacculifer* under low-pH treatments was significantly reduced. Until now, there has been no study reporting on the relationship between planktic foraminiferal size and pH in the water column. The present study provides the first attempt to link the individual size of planktic foraminifera with seawater pH. Our results from both the individual experiment and community culture indicated that a low pH slowed *T. sacculifer* growth and led to small individuals.

The changes in shell weight under different seawater pH conditions reflect the variations of calcification ability. This means that the planktic foraminifera may reduce the energetic costs of calcification under the stress of low pH [27], and build thin and small shells [7,8]. In the present study, after a period of culture, the final weight of the planktic foraminiferal shells decreased as the pH declined. This highlights the clear vulnerability of calcification when subjected to low pH. Many studies have confirmed that planktic foraminiferal calcification decreases under more acidic seawater conditions [8,28,29]. In addition, a decline in seawater pH may affect the physiological processes of foraminifera by affecting the symbiotic algae. Unfortunately, in the present experiments, we only focused on the individual biology of foraminifera, and the measurement of symbiotic algae was not fully considered. Future works are required in order to address this issue.

Additionally, existing studies observed a thinner shell in low-pH conditions [7,8]. However, due to challenging sea conditions, we were unable to measure the thickness of the shells in this study. Given that both their length and weight may vary in the growth process, we recommend using the ratio of weight/length (W/L) to reconstruct the seawater pH. Another potential issue is that the seawater carbonate system parameters (e.g., CO_3_^2−^, DIC, TA, etc.) were not monitored, due to the limitation of on-board culture conditions. Seawater pH and CO_3_^2−^ were found to be correlated, and had similar effects on the shell thickness, size, and SNW of planktic foraminifera [21]. The present study focused only on the variation in seawater pH, and the results were similar to those of previous studies.

Compared with the quantitative indices, such as shell size and weight, the biological processes of planktic foraminifera—such as locomotion ability, feeding behavior, and individual vitality—showed a more direct and intrinsic response to alterations in pH. *T. sacculifer* are representative of warm/oligotrophic spinose foraminifera. The variation of pseudopodia reflects the locomotion and feeding ability of planktic foraminifera [30]. Our results showed an interesting phenomenon, in that pseudopodia were lessened or lost under low-pH conditions (Figure 3), which adversely affected locomotion and feeding. Unlike previous studies [1,31], in which dead *Artemia nauplius* were used in the culture experiment, live *Artemia nauplius* that were incubated on-board were used in this study to assess the feeding ability of *T. sacculifer.* Additionally, we altered the feeding frequency from every other day in a previous study [32] to daily in the present study, creating more feeding opportunities for planktic foraminifera. Therefore, even though it was a qualitative observation of feeding behavior, our results confirmed that the feeding behavior of living planktic foraminifera decreased as the pH declined. To the best of our knowledge, the present study provides the first available data on the feeding behavior of living planktic foraminifera under different pH conditions. In future studies, assessing the quantitative effects of low pH on planktic foraminiferal feeding would be helpful to further quantify the potential effects of seawater pH decline on marine food webs.

The present study offered the first set of morphological indices with the potential to monitor the decline in seawater pH. The results also showed that the cytoplasmic vitality decreased as pH declined. A biological study showed that calcification, including the repair of the shell and pseudopodia, was closely related to cytoplasmic activity [30]. Therefore, the present study indicates that the live planktic foraminifera underwent a cascade biological response as the seawater pH declined. The acidified seawater reduces the vitality of the cytoplasm, as a result of which the activity of pseudopodia is weakened, leading to a reduction in locomotion and feeding, and a decrease in growth and calcification.

### 4.2. Paleoceanographic Significance of the Biological Responses of Symbiont-Bearing Calcifiers to Seawater pH

A key observation from the present study is that *T. sacculifer* were bleached under low-pH conditions (Figure 2). This phenomenon is a new observation in living planktic foraminifera. Previous studies only reported bleaching effects on sessile taxa—i.e., corals or large benthic foraminifera—and speculated that extreme environmental changes, such as high temperatures and solar radiation stress, may kill symbiotic dinoflagellates, and lead to cytoplasmic color change and rapid bleaching in these organisms [33,34,35,36]. The present study presents the first observations of the whole process of bleaching in living planktic foraminifera under low-pH conditions, which might reveal a new regulatory mechanism between seawater pH decline and marine calcifier bleaching. We postulate that, under the stress of low pH, planktic foraminifera may have a lower metabolism, and that they do not require symbionts. As a result, the symbionts disappear and the bleaching phenomenon occurs.

A few low-seawater-pH events similar to the situation forecasted for the future have occurred in the past (e.g., the PETM extreme event) [3,5,37]. *T. sacculifer* has a long evolutionary history, and first appeared with symbionts 23 million years ago, when the seawater pH (~8.00) was recovering and rising from the last low-seawater-pH event [38]. Later, symbiont-bearing species appeared in large numbers [16]. There is little doubt that favorable ecological conditions—for example, the pH conditions illustrated by the present study—can facilitate the occurrence of these species, and provide conditions under which they are able to thrive. The present study demonstrated that low pH led to bleaching in *T. sacculifer*, which suggests that seawater pH is an important factor for the evolution of symbionts in planktic foraminifera.

Additionally, *T. sacculifer* experiences periodic changes in depth (~0–100 m), with reproduction occurring on a synodic lunar cycle [19,39]. The disappearance of symbionts is linked to gametogenesis, during which gametes are released and the mother cells die—a point at which symbionts are no longer necessary. Reconstructed ocean pH–depth profiles showed that pH declined from ~8.10 to ~7.50 at ~100 m in the equatorial Pacific region [20]. Based on the experimental results, we speculate that the disappearance of symbionts when gametes are formed may be essentially regulated by a decline in seawater pH; the actual mechanism needs to be explored in future studies. As for certain benthic foraminifera, they could actively elevate their intracellular pH to promote calcification through proton pumping, and lower their extracellular pH [40]. Although no studies have previously been conducted regarding planktic foraminifera, we suspect that similar functions exist in planktic foraminifera—that is, symbiont-bearing planktic foraminifera could actively modify the pH in an ambient environment in order to diminish or acquire symbionts. Furthermore, based on the present study, we speculate that the loss of symbionts in planktic foraminifera might be an adaptive strategy adopted in response to low-pH stress. Once exposed to adverse environmental conditions (e.g., low pH), the host foraminifera could release symbionts and use the limited energy only in their own biological processes.

## 5. Conclusions

In this study, we carried out a series of on-board experiments with different pH gradients, ranging from 7.20 to 8.65, in order to evaluate the effect of seawater pH on the biological development and behavior of living *T. sacculifer*. Their survival time and growth rate in terms of length and weight consistently decreased as the pH declined. In addition, the cytoplasmic vitality also reduced, resulting in a decline in feeding. Equations between biological indices (length and weight) and seawater pH were therefore built, and could be used in paleoceanographic pH reconstruction. The phenomenon of bleaching (loss of symbionts and pseudopodia) in living planktic foraminifera under acidic conditions was first observed in culture experiments. Based on the present study, we postulate that the disappearance of symbionts, as a result of a decline in pH, leads to bleaching. This study provides a new perspective on the evolution of symbionts in planktic foraminifera in relation to seawater pH, in that perhaps the favorable seawater pH in a geological period (e.g., the increasing pH of the late Oligocene) drives the emergence of the symbiont-bearing species. Our findings imply that an ongoing decline in seawater pH may result in smaller individuals and reduced ingestion and shell weight of calcareous marine organisms.

## Figures and Tables

**Table 1:** Equations between individual biological indices (growth rate, length, weight, and W/L) and seawater pH, based on culture experiments.

*Trilobatus sacculifer*		r^2^	*p*-Value	Number of Specimens	Equations
Growth rate (G)	pH	0.97	0.0147	23	pH = (G + 137.21 (±18.20))/(19.42 ± 2.38)
Length (L)		0.88	0.0181	39	pH = (L + 726.26 (±201.19))/(118.08 ± 25.08)
Weight (W)		0.89	0.0159	39	pH = (W + 40.09 (±9.40))/(5.84 ± 1.18)
Weight/Length (W/L)		0.88	0.0186	39	pH = (W/L + 0.0096 (±0.0021))/(0.0479 ± 0.0167)

Note: All of the equations were transformed from the linear regression presented in Figure 1, Figure 4 and Figure 5.

## Data Availability

All data were available in supplementary materials.

## Supplementary Materials

The following are available online at https://www.mdpi.com/article/10.3390/biology11010098/s1: Figure S1: The in situ temperature, salinity, and light intensity data under different depths; Figure S2: The setup of the incubator culture program, view of the situation inside the incubator, and the light intensity instructions of the incubator; Figure S3: Representative fluorescent-labeled specimens from the community experiment; Figure S4: Survival time and growth rate of *Trilobatus* *sacculifer* under different pH treatments; Figure S5: Mean survival number (L_x_) and estimated life expectancy (e_x_) of *T. sacculifer* under different pH treatments and culture times; Figure S6: Length and weight of *T. sacculifer* cultured for 12 days at 6 different pH levels; Table S1: The original dataset of in situ temperature, salinity, and light intensity under different depths; Table S2: Detailed description of the individual experiments; Table S3: Life table of *T. sacculifer* cultured at 6 different pH levels; Table S4: Parameters of length measured from *T. sacculifer* cultured at 6 different pH levels; Table S5: Parameters of weight measured from *T. sacculifer* cultured at 6 different pH levels.
