# Supplementary material for: Biological Response of Planktic Foraminifera to Decline in Seawater pH
Source: Biology (Basel). 2022 Jan 9;11(1):98. doi: 10.3390/biology11010098 (PMC8773009; doi:10.3390/biology11010098)
Supplement: Supplementary file 1 [file biology-11-00098-s001.zip › Supplement Figures and Tables.pdf]

Supporting information

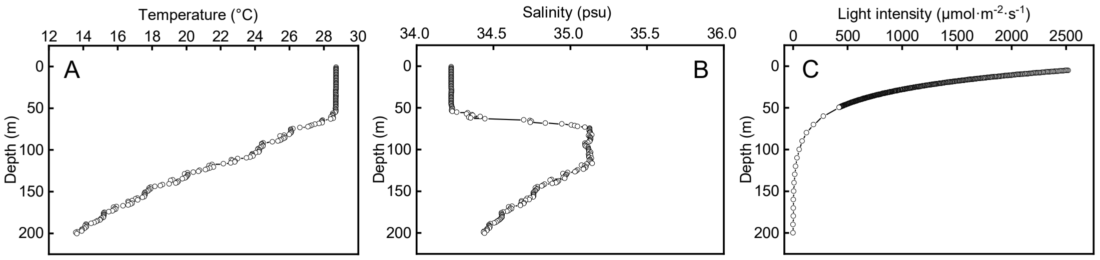

Figure S1. The in situ temperature (**A**), salinity (**B**), and light intensity (**C**) data under different depths.

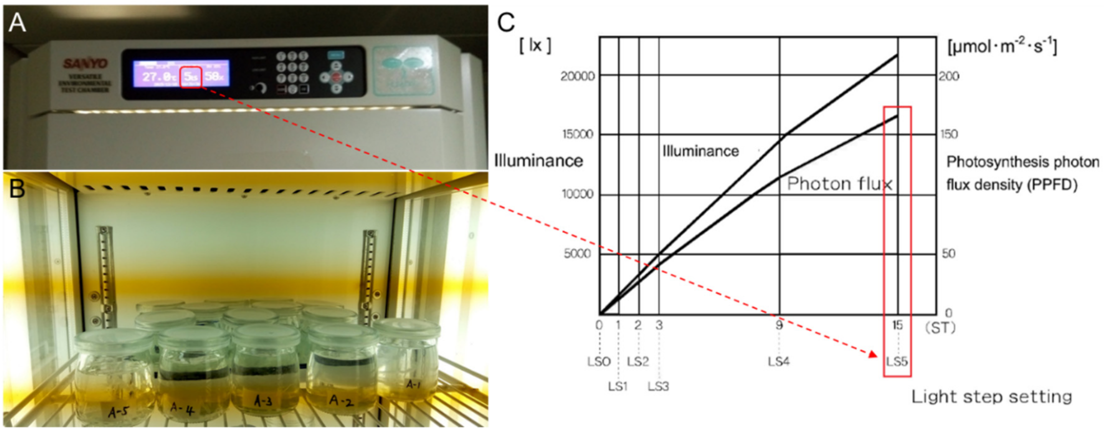

Figure S2. The setup of the incubator culture program (**A**), view of the situation inside the incubator (**B**), and the light intensity instruction of the incubators (**C**). Note: There were six levels of light intensity (LS0–LS5) for the incubator. In present study, we set the maximum level of light intensity (LS5, ~ 170 μmol·m^{-2}·s^{-1}/20000 Lux) and a 12h: 12h light: dark cycle at 27°C. The red boxes showed the light set in our experiment and the light intensity was about 170 μmol·m^{-2}·s^{-1}. Picture C was modified from the instruction manual for SANYO MLR-351H.

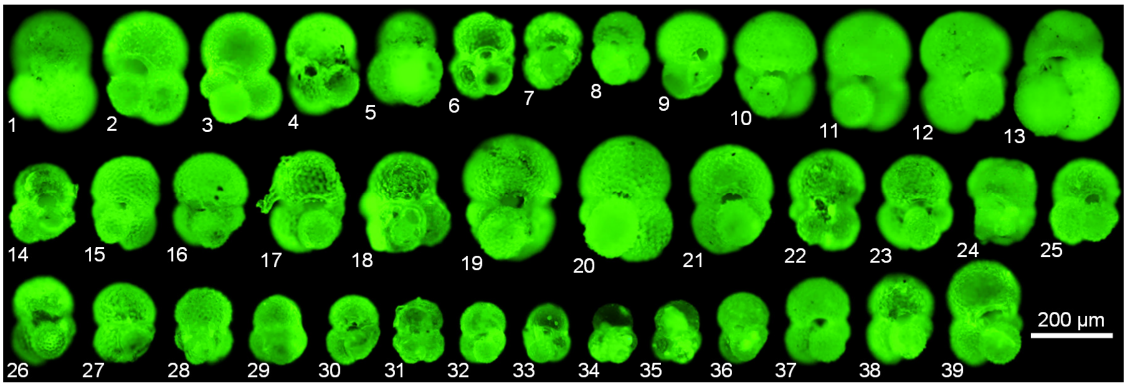

Figure S3. Representative fluorescent-labeled specimens from the community experiment. Note: pH 8.65: 1−6; pH 8.40: 7−13; pH 8.15: 14−25; pH 7.90: 26−33; pH 7.65: 34−39. Scale bar = 200 μm.

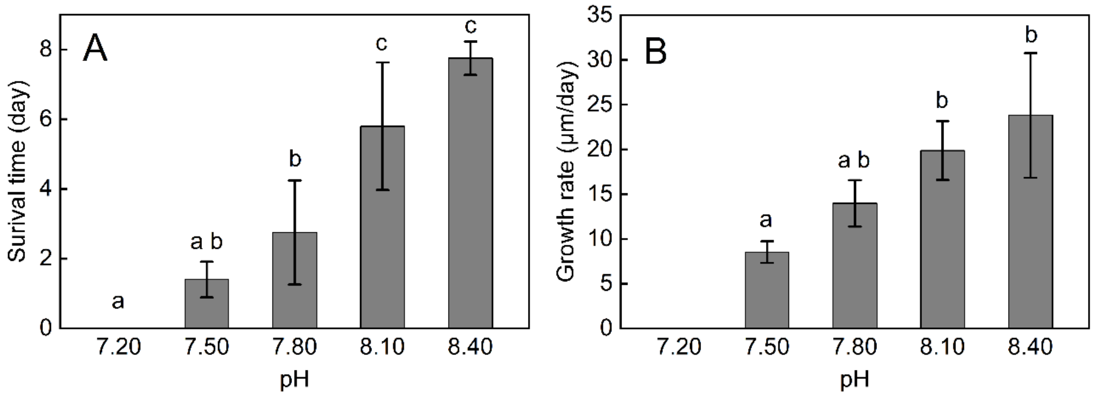

Figure S4. Survival time (**A**) and growth rate (**B**) of *Trilobatus sacculifer* under different pH treatments. Different letters on the top of histogram bars indicate significant differences ($p < 0.05$) from the analysis of ANOVA. Error bar = SE. Note: All individuals of *T. sacculifer* cultured at pH = 7.20 died within the first day, the mean survival time was zero and analyzed in the ANOVA. However, the growth rate couldn't be calculated and not analyzed in the ANOVA.

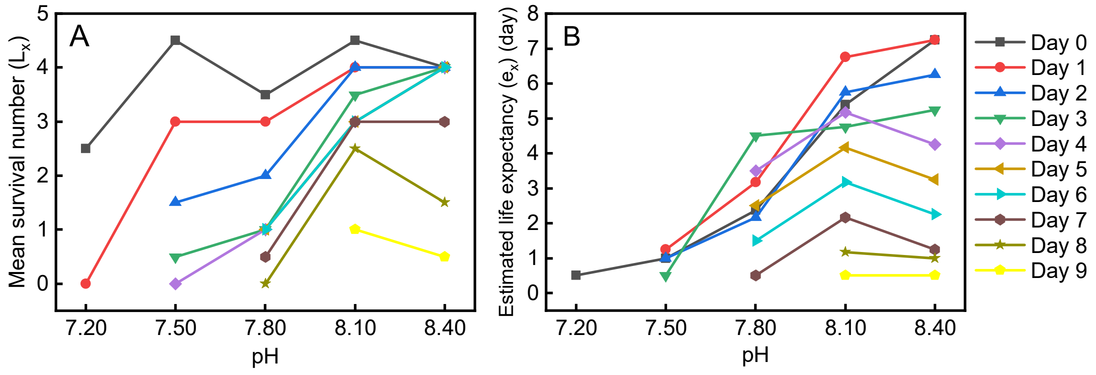

Figure S5. Mean survival number (L_x) (**A**) and estimated life expectancy (e_x) (**B**) of *T. sacculifer* under different pH treatments and culture times.

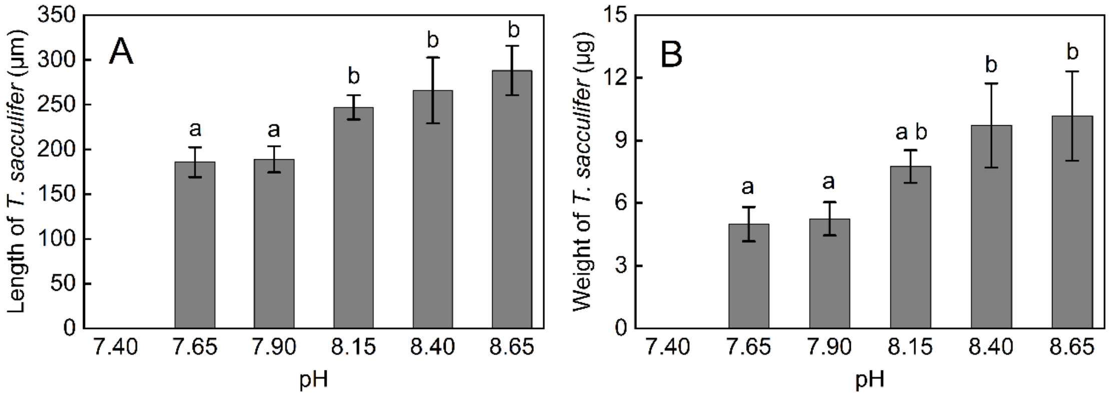

Figure S6. Length (**A**) and weight (**B**) of *T. sacculifer* cultured for 12 days at 6 different pH levels. Different letters on the top of histogram bars indicate significant differences ($p < 0.05$) from the analysis of ANOVA. Error bar = SE. Note: There was no living *T. sacculifer* under the pH 7.40 treatment and no data from this treatment was analyzed in the ANOVA.

Table S1. The original data-set of *in situ* temperature, salinity, and light intensity under different depths. It is a data set and uploaded separately in an excel file named as “Supporting Table S1”.

Table S2. Detailed description of the individual experiments.

Experimental set up	pH	Number of replicates (individuals)
Low pH	7.20	5
Low pH	7.50	5
Low pH	7.80	4
Reference	8.10	5
High pH	8.40	4

Table S3. Life table of *T. sacculifer* cultured at 6 different pH levels. Note: The first column (x) shows the age and the second column (N_x) is the number of living *T. sacculifer* at the beginning of each age stage. The third column (l_x) is the proportion of surviving comparing the initial number at each age stage. The forth column (d_x) is the number of dying individual at each age stage. The fifth column (q_x) was the ratio of the individual dying at each age stage. The sixth and seventh columns are two parameters and calculated as $L_x = (l_x + l_{x+1})/2$, $T_x = L_x + L_{x+1} + \cdots + L_{x+n}$. The eighth column ($e_x$) is the life expectancy and calculated as $e_x = T_x/N_x$.

Treatments	Age (x)	N_x	l_x	d_x	q_x	L_x	T_x	e_x
	0	5	1	5	1	2.5	2.5	0.5

pH 7.20	1	0	0	−	−	0	0	−
pH 7.50	0	5	1	1	0.2	4.5	5	1
	1	4	0.8	2	0.5	3	5	1.25
	2	2	0.4	1	0.5	1.5	2	1
	3	1	0.2	1	1	0.5	0.5	0.5
	4	0	0	−	−	0	0	−
pH 7.80	0	4	1	1	0.25	3.5	9.5	2.38
	1	3	0.75	0	0	3	9.5	3.17
	2	3	0.75	2	0.66	2	6.5	2.17
	3	1	0.25	0	0	1	4.5	4.5
	4	1	0.25	0	0	1	3.5	3.5
	5	1	0.25	0	0	1	2.5	2.5
	6	1	0.25	0	0	1	1.5	1.5
	7	1	0.25	1	1	0.5	0.5	0.5
	8	0	0	−	−	0	0	−
pH 8.10	0	5	1	1	0.2	4.5	27	5.4
	1	4	0.8	0	0	4	27	6.75
	2	4	0.8	0	0	4	23	5.75
	3	4	0.8	1	0.25	3.5	19	4.75
	4	3	0.6	0	0	3	15.5	5.17
	5	3	0.6	0	0	3	12.5	4.17
	6	3	0.6	0	0	3	9.5	3.17
	7	3	0.6	0	0	3	6.5	2.17
	8	3	0.6	1	0.33	2.5	3.5	1.17
	9	2	0.4	2	1	1	1	0.5
	10	0	0	−	−	0	16	−
pH 8.40	0	4	1	0	0	4	29	7.25
	1	4	1	0	0	4	29	7.25
	2	4	1	0	0	4	25	6.25
	3	4	1	0	0	4	21	5.25
	4	4	1	0	0	4	17	4.25
	5	4	1	0	0	4	13	3.25
	6	4	1	0	0	4	9	2.25
	7	4	1	2	0.5	3	5	1.25
	8	2	0.5	1	0.5	1.5	2	1
	9	1	0.25	1	1	0.5	0.5	0.5
	10	0	0	−	−	0	0	−

Table S4. Parameters of length measured from *T. sacculifer* cultured at 6 different pH levels.

pH	7.40 (N=0)	7.65 (N=6)	7.90 (N=8)	8.15 (N=12)	8.40 (N=7)	8.65 (N=6)
Length (μm)	—	185.86 ± 16.64	189.02 ± 14.65	247.01 ± 13.56	265.83 ± 36.73	288.15 ± 27.44

Table S5. Parameters of weight measured from *T. sacculifer* cultured at 6 different pH levels.

pH	7.40 (N=0)	7.65 (N=6)	7.90 (N=8)	8.15 (N=12)	8.40 (N=7)	8.65 (N=6)
Weight (μg)	—	5.00 ± 0.82	5.25 ± 0.80	7.75 ± 0.79	9.71 ± 2.02	10.17 ± 2.14
